# U87MG glioma cells overexpressing IL-17 acclerate early-stage growth *in vivo* and cause a higher level of CD31 mRNA expression in tumor tissues

**DOI:** 10.3892/ol.2013.1518

**Published:** 2013-08-07

**Authors:** JINHUI HU, HONGXING YE, DENGHAI ZHANG, WEIWEI LIU, MIN LI, YING MAO, YUAN LU

**Affiliations:** 1 Department of Lab Medicine, Gongli Hospital, Second Military Medicine University, Pudong New Area, Shanghai 200135, P.R. China; 2 Department of Lab Medicine, Huashan Hospital, Fudan University, Shanghai 200040, P.R. China; 3 Department of Neurosurgery, Huashan Hospital, Fudan University, Shanghai 200040, P.R. China; 4 State Key Laboratory of Medical Neurobiology, Fudan University, Shanghai 200032, P.R. China

**Keywords:** glioma, IL-17, pEGFP-N1, tumorigenesis, nude mice

## Abstract

Immunological alterations have been reported to be involved in glioma, the most common malignant disease of the adult brain. Our recent study identified higher levels of IL-17 in glioma specimens. The present study investigated the role and possible mechanisms of IL-17 in glioma tumorigenesis. Human IL-17 cDNA was cloned and inserted into the eukaryotic pEGFP-N1 expression vector, which was used to transfect the glioma U87MG cell line, resulting in a high level of IL-17 expression in these cells. The cells were then transfected with IL-17 (pEGFP-N1-IL-17-U87MG) or mock (pEGFP-N1-U87MG) vector or left untransfected (U87MG) and subcutaneously inoculated into the right flank of nude mice. The results revealed that the pEGFP-N1-IL-17-U87MG cells grew more rapidly in the early stages (P<0.05, determined on day 32 post-inoculation compared with the other two groups). Quantitative (q)PCR detected higher mouse (m)CD31 mRNA levels in the IL-17-transfected group (P<0.01) compared with the mock-transfected and untransfected groups. IL-17 transfection altered the mRNA expression of a panel of molecules that are associated with immunity and inflammation in U87MG cells *in vitro*. An effect of the vector was identified, whereby the mock transfection strongly inhibited cell growth *in vivo* and dramatically altered the mRNA levels of multiple molecules in the cell culture *in vitro* compared with the untransfected cells. The present study confirmed that IL-17 overexpression may enhance glioma cell growth *in vivo*, which may be associated with accelerated angiogenesis. IL-17 overexpression may also alter the cellular mRNA expression of immune-related molecules.

## Introduction

Glioma is the most common malignant disease of the adult brain. The outcome of patients with glioma is poor, mainly due to the diffusion of the tumor into the brain parenchyma (1). Certain immunological dysfunctions have been identified in glioma, including elevated immunosuppressive factors (2–5), reduced total lymphocytes (6), an imbalance in T helper (Th) subsets (7–14) and the infiltration of immunosuppressive microglia and macrophage cells (15). The immunosuppressive mechanism causes patients to be incapable of eradicating tumor cells and results in the anergy of certain immunotherapies. Therefore, the identification of the role of immune regulatory factors in glioma is significant for obtaining an understanding of the tumorigenesis mechanism and identifying a new therapeutic strategy for this malignant disease.

IL-17 is a main effecter cytokine of Th17 cells and has become a topic of interest following the identification of Th17 in immunology (16,17). IL-17 has been examined in immunology, including autoimmunity, infection, transplantation, allergy and tumors. IL-17 has been shown to promote tumorigenesis via certain mechanisms, including the upregulation of angiogenesis-related molecules, vascular endothelial growth factor (VEGF) and CD31, the activation of the IL-6-STAT3 signaling pathway, the downregulation of IL-12Rβ2, thus impairing Th1 function, and the suppression of cytotoxic T lymphocytes (CTLs), causing them to lose their cytotoxic effect via co-operation with CD8 (18–20).

Our recent study identified that IL-17 was expressed at a higher level in glioma tissues compared with trauma tissues (21). Other studies have also demonstrated that IL-17 or Th17 are expressed at higher levels in glioma (22,23). To further explore the role and progress of IL-17 in glioma tumorigenesis, human IL-17 cDNA was cloned and packed into the eukaryotic pEGFP-N1 expression vector. The recombinant pEGFP-N1-IL-17 vector was then stably transfected and expressed in the glioma U87MG cell line. The present study investigated the role of IL-17 in promoting glioma tumorigenesis.

## Materials and methods

### Recombinant vector and gene amplification

The pEGFP-N1 plasmid was provided by the Institute of Military Medicine Science (Beijing, China) and re-confirmed by sequencing. Peripheral blood (2 ml) was drawn from the peripheral vein from a patient with idiopathic thrombocytopenic purpura (ITP) at the Huashan Hospital (Fudan University, Shanghai, China), according to the Sample Manipulation Guidelines of Huashan Hospital. This study was approved by the ethics committee of Huashan Hospital, Fudan University. Written informed consent was obtained from the patient. Peripheral blood mononuclear cells (PBMCs) were separated by Ficoll centrifugation at 400 × g. The PBMCs were cultured in RPMI-1640 medium supplemented with 100 μg/ml penicillin, 100 μg/ml streptomycin, 2 mM glutamine and 10% heat-inactivated fetal calf serum (Gibco, Carlsbad, CA, USA). The PBMCs were stimulated for 4 h with 50 ng/ml phorbol myristate acetate (PMA; Sigma-Aldrich, St Louis, MO, USA) and 1 μM ionomycin in the presence of 10 μg/ml brefeldin A (Alexis Biochemicals, San Diego, CA, USA). mRNA was extracted and cDNA was synthesized using quantitative (q)PCR with primers containing enzymatic digestion sites for *Bam*HI and *Sal*I, according to the manufacturer’s instructions. The primers corresponded to NCBI Reference Sequence (NM_002190.2) forward, 5′-CAG TCG ACG ATG ACT CCT GGG AAG ACC TCA TTG-′3 and reverse, 5′-GG TGG ATC CCG GGC CAC ATG GTG GAC AAT CGG-′3. The IL-17 cDNA was packed into a pMD^®^19-T Simple Vector (Takara, Otsu, Japan) to form the pMD19-T-IL-17 vector. Following the sequencing, the recombinant segment of the correct clone was incised by *Bam*HI and *Sal*I (Takara). The recombinant segment was packed into pEGFP-N1, which was incised by the same two restriction endonucleases. The pEGFP-N1-IL-17 clones were sequenced and the correct clones were amplified and identified by restriction enzyme digestion.

### Cell line and transfection

The human glioma U87MG cell line was purchased from Cell Bank (Shanghai Life Science Institute, Science Academy of China, Shanghai, China; ATCC no. HTB-14™). The cells were cultured in Dulbecco’s modified Eagle’s medium (DMEM) supplemented with 100 μg/ml penicillin, 100 μg/ml streptomycin, 2 mM glutamine and 10% heat-inactivated fetal calf serum (Gibco). The cells were then cultured at a density of 1×10^6^ cells/well in a 6-well plate, and 20 μg pEGFP-N1-IL-17 or pEGFP-N1 plasmid were transfected into the U87MG cells using Xfect reagent (Takara), according to the manufacturer’s instructions. At 24 h post-transfection, G418 was added to the culture medium (200 μg/ml). The cells were collected at 10 days post-transfection, when the majority of the cultured cells had died. The remaining cells were diluted to 1 cell/10 μl and 10 μl cells was added into the 96-well plate with G418. The cells were identified by fluorescence and the positive clones were transferred into a 6-well plate. Following amplification for 3 days, the cells were collected for mRNA extraction and qPCR detection (Takara). The following IL-17 primer was used: Forward, 5′-CTG AAC ATC CAT AAC CGG AAT ACC A-′3 and reverse, 5′-AGC GTT GAT GCA GCC CAA G-′3. In order to determine IL-17 secretion, 1×10^4^ cells were cultured for 3 days, the culture supernatants were collected and IL-17 was detected using an ELISA kit (R&D, Minneapolis, MN, USA), according to the manufacturer’s instructions.

### Mice and xenograft tumor inoculation

Nude mice were purchased and bred at the Animal Laboratory Center, Fudan University. All the mice were handled within a specific pathogen-free facility. All the experimental manipulations were undertaken in accordance with the Guidelines for the Care and Use of Laboratory Animals of Fudan University. This study was approved by the animal ethics committee of Shanghai Medical college, Fudan University. pEGFP-N1-U87MG, pEGFP-N1-IL-17-U87MG and U87MG cells (5×10^5^ cells of each type) were inoculated subcutaneously in the right flanks of the nude mice, with 10 mice in each group. The xenograft tumorigenesis effects were observed for the first time at 7 days post-inoculation and monitored once every 3 days. The tumor volumes were measured on days 32 and 35 post-inoculation. The mice were sacrificed on day 39 and the masses and volumes of the xenograft tumors and spleens were measured. Tumor volume (V) was calculated using the formula *V = ab*^2^/2 (a and b are the long and short diameters of the tumor, respectively). The RNA of the tumor tissue was extracted and mouse (m)-CXCR2, -CD31, -matrix metalloproteinase 3 (MMP3) and -intercellular adhesion molecule-1 (ICAM-1) were qualified using qPCR. The conditions for the qPCR were 95°C for 30 sec, 95°C for 10 sec and 60°C for 20 sec for 40 repeats and 95°C-60°C-95°C for the melt curve observation. The primers that were used are listed in Table I. Each sample was tested in triplicate and the RNA of the target molecules were normalized to glyceraldehyde 3-phosphate dehydrogenase (GAPDH). The results were calculated as 2^−ΔΔCT^.

### qPCR determination of mRNA expression in vitro

In addition to using qPCR to detect the four mouse genes being expressed in the tumor tissues, the mRNA of a panel of molecules associated with immune and inflammatory responses in cells cultured *in vitro* were also detected using this technology. The molecules that were detected were: The chemokines, CXCL1, CXCL5, CXCL8, CXCL10, CXCL11, monocyte chemoattractant protein-1 (MCP-1; CCL2), regulated on activation, normal T cell expressed and secreted (RANTES; CCL5), CCL20, CCR4 and CCR6; the immunology regulation factors, β2-MG, PD-L1, prostaglandin E2 (PGE2), transforming growth factor (TGF)-β, IL-6 and STAT3; and the intercellular matrix molecules, MMP3, ICAM-1 and VEGF. The primers for the detection of these genes were human sequence-specific while those that were used for the detection of the genes in the tumor tissues were mouse-specific (with ‘m’ prefixing the gene name in this study). The primer sequences are listed in Table I. qPCR was performed using the conditions that were described previously.

### Statistical analysis

The statistical analysis was performed using SPSS 11.5 (SPSS, Inc., Chicago, IL, USA). The data were analyzed by ANOVA and logarithmic transformation was used if necessary. P<0.05 was considered to indicate a statistically significant difference.

## Results

### Human IL-17 is successfully expressed in U87MG glioma cells

**Figure 1:**
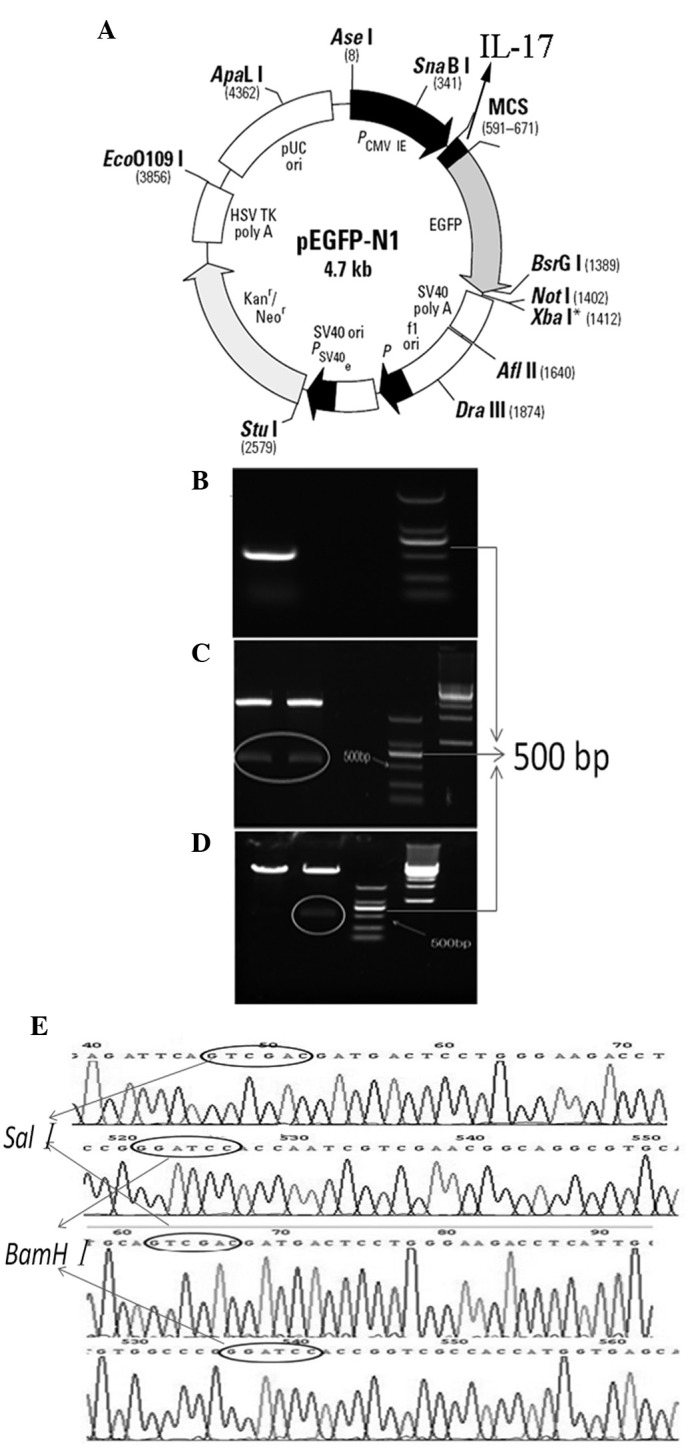
Synthesis of IL-17 cDNA and construction of the pEGFP-N1-IL-17 plasmid. (A) IL-17 cDNA was cloned into the MCS site of pEGFP-N1. (B) PCR for IL-17 cDNA. mRNA was extracted from the PBMCs of an ITP patient and reverse transcribed to cDNA, from which IL-17 was amplified. (C and D) Restriction enzyme digestion of pMD^®^19-T-IL-17 and pEGFP-N1-IL-17 recombinant plasmids showing the IL-17 fragment with 468 bp. (E) Sequencing of the pMD19-T-IL-17 and pEGFP-N1-IL-17 recombinant plasmids. *Sal*I and *Bam*HI recognition sequences were identified and the sequence between them fully corresponded to IL-17 from Genbank (accession no. NM_002190.2). MCS, multiple cloning site; PBMC, peripheral blood mononuclear cells; ITP, idiopathic thrombocytopenic purpura.

IL-17 cDNA (NCBI reference sequence, NM_002190.2) was synthesized using RNA that was extracted from PBMCs of an ITP patient. Sequencing and restriction enzyme digestion were used to confirm the successful packages of IL-17 cDNA into the pMD19-T and pEGFP-N1 vectors. The results revealed that the IL-17 cDNA was inserted into the multiple cloning site (MCS) of the pEGFP-N1 vector. The target gene fragment (IL-17 cDNA) was 468bp and the sequence fully corresponded to that in Genbank (Fig. 1).

**Figure 2:**
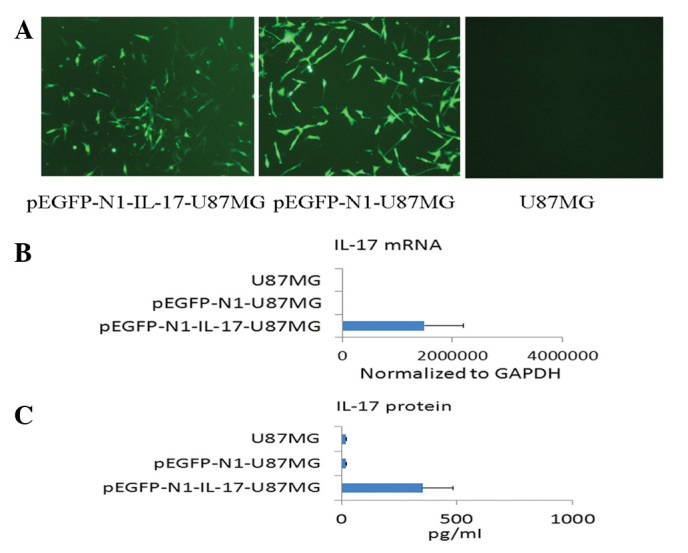
Identification of stably-transfected pEGFP-N1-IL-17-U87MG cells. (A) Fluorescence detection of expanded cultured cells from a monoclone. Cells that were transfected with pEGFP-N1-IL-17-U87MG and pEGFP-N1-U87MG exhibited a bright fluorescence, while the untransfected U87MG cells demonstrated no fluorescence. IL-17 (B) mRNA and (C) protein level detection in the three cell lines. The pEGPF-N1-IL-17-U87MG cells presented higher IL-17 mRNA levels in the cell extracts and higher protein levels in the supernatant than the pEGFP-N1-U87MG and U87MG (P<0.001) cells. When normalized to GAPDH, the IL-17 mRNA levels of the pEGPF-N1-IL-17-U87MG, pEGPF-N1-U87MG and U87MG cells were 1487588±708137, 1.28±0.47 and 1.03±0.21, respectively. The IL-17 protein levels in the supernatants of the pEGPF-N1-IL-17-U87MG, pEGPF-N1-U87MG and U87MG cells were 351.6±130, 17.3±3.6 and 17.4±3.5 pg/ml, respectively. GAPDH, glyceraldehyde 3-phosphate dehydrogenase.

The cells that were transfected with pEGFP-N1-IL-17 and pEGFP-N1 were selected using 200 μg/ml G418. Following 10 days, the cells were diluted to 1 cell/10 μl. Subsequent to forming an expansive culture, the cells were identified using a fluorescence microscope and IL-17 mRNA and protein expression was detected by qPCR and an enzyme-linked immunosorbent assay (ELISA). The results revealed that the U87MG cells that were transfected with pEGFP-N1-IL-17 and pEGFP-N1 exhibited fluorescence, indicating that the vector was expressed successfully in those cells. Notably, the pEGFP-N1-IL-17-U87MG cells demonstrated a significantly higher level of IL-17 mRNA and protein compared with the pEGFP-N1-U87MG and U87MG cells (P<0.001; Fig. 2).

### IL-17 overexpression promotes U87MG tumorigenesis in nude mice with elevated CD31 in tumor tissues

**Figure 3:**
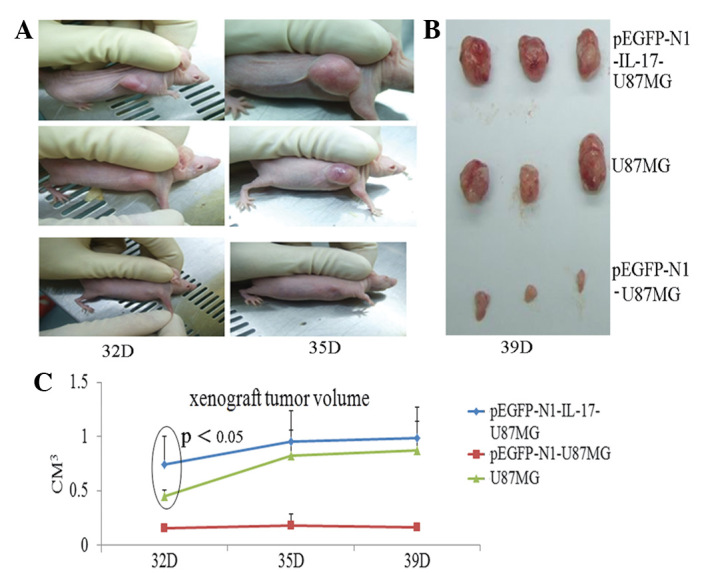
Differential tumorigenesis effect of pEGFP-N1-IL-17-U87MG, pEGFP-N1-U87MG and U87MG cells. (A) At 32 days post-xenograft, the tumor volumes of the pEGFP-N1-IL-17-U87MG mice were higher than those of the U87MG mice (P<0.05). At days 35 and 39, the tumor volume remained higher, but lacked statistical significance. Only 6 out of the 10 pEGFP-N1-U87MG mice presented with tumors and the tumor growth rate was slower than of the other two groups at all the time points (P<0.01). (A) Mice at 32 and 35 days; (B) tumors at 39 days; (C) statistics of the tumor volume at days 32–39.

pEGFP-N1-IL-17-U87MG, pEGFP-N1-U87MG and U87MG cells (5×10^5^) were subcutaneously inoculated into the right flanks of the nude mice. At 7 days post-inoculation, neoplasms became visible and the tumor sizes were monitored every 3 days. At 32 days post-inoculation, the sizes of the neoplasms in the pEGFP-N1-IL-17-U87MG group were larger than those of the pEGFP-N1-U87MG (P<0.05) and U87MG (P<0.05) groups. At 35 and 39 days, the tumor volume of the former group remained larger than the latter two groups, but had no statistical significance with the U87MG group, indicating that IL-17 may have accelerated tumor growth at an early stage (Fig. 3).

**Figure 4:**
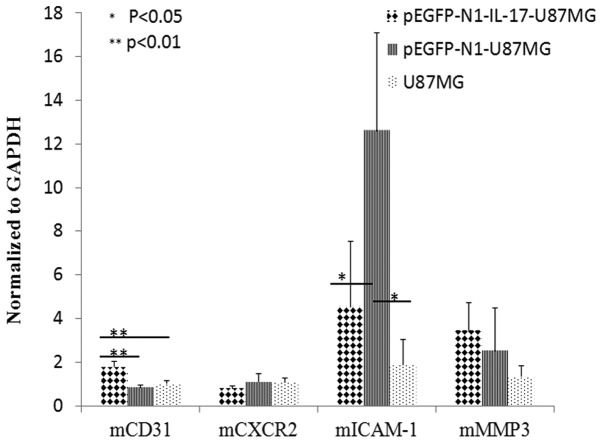
Variation of angiogenesis-related molecules in the xenografted tumors of the pEGFP-N1-IL-17-U87MG, pEGFP-N1-U87MG and U87MG cells. mCD31 mRNA levels were higher in the tumor tissues of the pEGFP-N1-IL-17-U87MG mice than in the other two groups (P<0.01). mICAM-1 mRNA levels were higher in the tumor tissues of the pEGFP-N1-U87MG mice than in the other groups (P<0.05). GAPDH, glyceraldehyde 3-phosphate dehydrogenase; mICAM-1, mouse intercellular adhesion molecule-1; mMMP3, mouse matrix metalloproteinase 3.

To explore the possible mechanism underlying the differences in tumor growth among the three groups, the mRNA levels of mCXCR2, mICAM-1, mMMP3 and mCD31 were detected in the tumor tissues. The results revealed a higher mCD31 mRNA level in the pEGFP-N1-IL-17-U87MG group (P<0.01) compared with the other two groups, while mICAM-1 mRNA was higher in the pEGFP-N1-U87MG group (P<0.05) compared with the other two groups. The levels of mCXCR2 and mMMP3 mRNA were not significantly different among the three groups (Fig. 4).

### IL-17 transfection alters the mRNA levels of a panel of immune/inflammation-related molecules in U87MG

**Figure 5:**
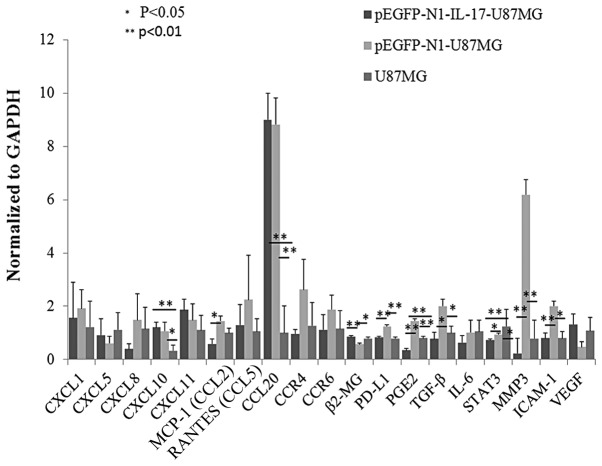
Variation of a panel of immune system- and inflammation-related molecules between pEGFP-N1-IL-17-U87MG, pEGFP-N1-U87MG and U87MG cells. The pEGPF-N1-IL-17-U87MG cells exhibited higher CXCR10 and CCL20 mRNA levels than the U87MG cells (P<0.01) and higher β-2MG mRNA levels than the pEGFP-N1-U87MG cells (P<0.01). The pEGPF-N1-U87MG cells expressed higher CXCL10, TGF-β, ICAM-1 (P<0.05), CCL20, PD-L1, PGE2 and MMP3 (P<0.01) mRNA levels than the U87MG cells and higher MCP-1, TGF-β, STAT3 (P<0.05), PD-L1, PGE2, MMP3 and ICAM-1 levels (P<0.01) than the pEGPF-N1-IL-17-U87MG cells. The U87MG cells expressed higher β-2MG mRNA levels than the pEGPF-N1-U87MG cells (P<0.05) and higher STAT3 levels than the pEGPF-N1-IL-17-U87MG cells (P<0.01). Each group consisted of 6 independent samples, each of which had been tested in triplicate. TGF-β, transforming growth factor-β; ICAM-1, intercellular adhesion molecule-1; MMP3, matrix metalloproteinase 3; MCP-1, monocyte chemoattractant protein-1; VEGF, vascular endothelial growth factor; RANTES, regulated on activation, normal T cell expressed and secreted (CCL5); PGE2, prostaglandin E2.

To further understand the role of IL-17 in the behavior of U87MG cells, qPCR was used to detect the mRNAs for a panel of proteins that are associated with immune and inflammation responses, including intercellular adhesion, the intercellular matrix and chemokines. The expression of a series of molecules was altered in the pEGFP-N1-IL-17-U87MG, pEGFP-N1- U87MG and U87MG cells (Fig. 5).

## Discussion

IL-17, as the main regulatory element of the emerging Th17 subset, has gained considerable interest. Our recent study identified a higher level of IL-17 in glioma tissue (21). The present study identified that the overexpression of IL-17 may accelerate the early-stage growth of U87MG glioma cells *in vivo*. The expression of Il-17 also altered the mRNA profile of immune/inflammation-related proteins when transfected into a cell culture *in vitro*.

In the present study, human IL-17 cDNA was inserted into the pEGFP-N1 plasmid and transfected into the glioma U87MG cell line. The success of the procedure was confirmed using gene sequencing, GFP detection and IL-17 mRNA and protein determination. The U87MG, pEGFP-N1-U87MG and pEGFP-N1-IL-17-U87MG cells were inoculated into nude mice. The pEGFP-N1-IL-17-U87MG group demonstrated accelerated tumorigenesis compared with the other two groups when measured at 32 days post-inoculation (P<0.05). On days 35 and 39 post-inoculation, the implanted tumors of the pEGFP-N1-IL-17-U87MG group were larger than those of the other two groups. However, the difference was not statistically significant. This result indicated that IL-17 was able to accelerate glioma growth, particularly in the early stage of tumorigenesis.

To identify the possible mechanism behind the accelerated tumor growth caused by IL-17 overexpression, mCXCR2, mMMP3, mICAM-1 and mCD31 mRNA expression in the xenografted tumor tissues were analyzed using qPCR. mCD31 expression in the tumor tissues of the pEGFP-N1-IL-17-U87MG group was higher than in the other groups (P<0.01). This was consistent with the results of a study by Numasaki *et al*(24). The effect of early-stage tumorigenesis acceleration caused by the overexpression of IL-17 may be associated with the promotion of angiogenesis, which is consistent with the notion that the formation of new blood vessels is vital for the initial growth stage for solid tumors. However, in contrast with the results from the study by Numasaki *et al*, the present data did not include an elevation in mCXCR2 in the tumor tissues from the IL-17 overexpression group. This difference may have been due to the different tumor types that were used in the two studies. Alternatively, the angiogenesis-promoting effects of IL-17 may also be active through pathways other than CXCR2, as described by a number of studies (25–27). The present study highlights the fact that IL-17 may be a target for interference in tumor angiogenesis.

In addition to detecting the mRNA levels of several molecules in the tumor tissues, the mRNA level for a panel of molecules that are associated with the immune response and inflammation were also analyzed in the transfected cells in order to understand the alterations caused to the behavior of the U87MG cells by IL-17 *in vivo*. IL-17 was able to increase the levels of CXCL10, CCL20 and β2-MG. However, whether these changes are associated with the acceleration of the early-stage growth of the U87MG cells *in vivo* remains to be elucidated. Among the molecules that were studied, β2-MG was particularly significant, since it was the only one among the observed panel that showed an elevation in the IL-17-transfected cells compared with those that were mock-transfected. β2-MG was reported to be an early marker in the plasma of hepatocellular carcinoma patients (28). However, the association between β2-MG and glioma remains elusive and requires further investigation. In the present study, the IL-17-transfected cells contained elevated levels of VEGF mRNA, although they were not increased to a statistically significant level compared with the mock-transfected cells. This elevation of VEGF may be important, since IL-17 tumor-promoting effects are associated with enhanced angiogenesis, as suggested by the present study and others (24,29,30).

Another noteworthy observation in the present study was the effect of the vector; the pEGFP-N1 expression vector was able to dramatically alter the behavior of the U87MG cells *in vivo* and *in vitro*. The mock transfection group demonstrated a strong suppression of nude mice xenograft tumorigenesis. On days 32, 35 and 39 post-inoculation, the pEGFP-N1-U87MG group showed a slower rate of tumorigenesis than the other two groups (P<0.01). In addition, the tumor-inhibiting effects were also revealed by the fact that only six out 10 pEGFP-N1-U87MG mice developed tumors. It is noteworthy that this tumor-inhibitory effect of the vector highlights the tumor-promoting effect of IL-17, since the mock transfection group was a more valued control for the IL-17-transfected group compared with the untransfected group. The *in vitro* culture also demonstrated the effects of the vector, as there were relatively more molecules showing the alterations in the mRNA levels compared with the IL-17 transfection group. The underlying mechanism for the effect that was observed remains unknown and thus requires further investigation.

In the present study, human IL-17 cDNA was successfully constructed in the glioma U87MG cell line using eukaryotic pEGFP-N1 expression vectors. The pEGFP-N1-IL-17-U87MG cells demonstrated accelerated growth in the early stage subsequent to being inoculated into nude mice, which was accompanied with a higher CD31 expression, indicating that an angiogenesis-promoting action may be involved. IL-17 transfection may also alter the mRNA levels of certain molecules that are associated with the immune response and inflammation. The vector exhibited an effect in the mock transfection group, with suppressed tumor growth and altered mRNA levels of multiple molecules. The mechanism for this phenomenon requires further investigation. The present study revealed that IL-17 was able to enhance glioma growth and change the expression of certain genes.

## Figures and Tables

**Table I:** Primer sequences.

Gene	Forward primer	Reverse primer	Product size, bp
GAPDH	TTCGACAGTCAGCCGCATCT	GTGACCAGGCGCCCAATACG	115
MCP-1	GGCTGAGACTAACCCAGAAACATC	TGACTGGGGCATTGATTGCAT	158
RANTES	GCTGCTTTGCCTACATTGCCC	ACTTGGCGGTTCTTTCGGGTG	118
CXCL1	GAACGTGAAGTCCCCCGGAC	GCCACCAGTGAGCTTCCTCC	175
CXCL5	GCAGCGCTCTCTTGACCACT	ACGCAACGCAGCTCTCTCAA	169
CXCL8	AACTTTCAGAGACAGCAGAGCACAC	GCACTCCTTGGCAAAACTGCAC	173
CXCL10	TGAGCCTACAGCAGAGGAACCT	TGCTGATGCAGGTACAGCGTAC	139
CXCL11	GCCTTGGCTGTGATATTGTGTGC	CTGCTTTTACCCCAGGGCCT	94
CCL20	CAGTGCTGCTACTCCACCTCTG	TGCCGTGTGAAGCCCACAATAA	112
CCR4	CTTCCTGAGCAAGCCTGGCA	AGGCTCCTCAAGGCAGGTCT	101
CCR6	TATTGAGTCACCTCTACTTTCCT	ACTGGAGTCGAAAACATCGCTGA	147
PD-L1	TGGTGGTGCCGACTACAAGC	GGGTAGCCCTCAGCCTGACA	129
STAT3	AGGAGCATCCTGAAGCTGACCCA	GAGGGTTCAGCACCTTCACCATT	163
β2-MG	AGTATGCCTGCCGTGTGAACC	GCGGCATCTTCAAACCTCCAT	100
PGE2	TGTTTTGAATGGGCGCCCG	CGGGAACGTTTGCAGACCGT	173
IL-6	AAGCCAGAGCTGTGCAGATGA	TGGTTCTGTGCCTGCAGCTT	136
TGF-β	GCCGAGCCCTGGACACCAAC	GCGCCCGGGTTATGCTGGTT	220
ICAM-1	AGTCGACGCTGAGCTCCTCT	TGTCTGGGCATTGCCAGGTC	137
VEGF	GGTGCCCGCTGCTGTCTAAT	CGCCTCGGCTTGTCACATCT	194
MMP3	GCTAAGTAAAGCCAGTGGAAATGAA	ACAGGACCACTGTCCTTTCTCC	199
mCXCR2^a^	GTTCAACCAGCCCTGACAGCT	TGGCAGAATAGAGGGCATGCC	207
mCD31^a^	GGAAGCCAACAGCCATTACGG	GAGCCTTCCGTTCTCTTGGTGA	151
mMMP3^a^	GTGTGCTCATCCTACCCATTGC	TAGTGTTGGAGTCCAGCTTCCCT	211
mICAM-1^a^	TGGCCCTGCAATGGCTTCAA	AGTCTCCAAGCCCAGGCTGA	196

a Gene names that are preceded by ‘m’ indicate that the primer sequence is mouse-specific. Otherwise, the primers are human-specific.

GAPDH, glyceraldehyde 3-phosphate dehydrogenase; MCP-1, monocyte chemoattractant protein-1; RANTES, regulated on activation, normal T cell expressed and secreted (CCL5); PGE2, prostaglandin E2; TGF-β; transforming growth factor-β; VEGF, vascular endothelial growth factor; MMP, matrix metalloproteinase; ICAM-1, intercellular adhesion molecule-1.
